# Sunitinib in metastatic thymic carcinomas: Laboratory findings and initial clinical experience

**DOI:** 10.1038/sj.bjc.6605740

**Published:** 2010-06-22

**Authors:** P Ströbel, R Bargou, A Wolff, D Spitzer, C Manegold, A Dimitrakopoulou-Strauss, L Strauss, C Sauer, F Mayer, P Hohenberger, A Marx

**Affiliations:** 1 Institute of Pathology, University Medical Center Mannheim, University of Heidelberg, Theodor-Kutzer-Ufer 1-3, 68135 Mannheim, Germany; 2 Division of Hematology and Medical Oncology, Department of Internal Medicine II, University of Würzburg, Klinikstraße 6, 97070 Würzburg, Germany; 3 Institute of General Medicine, Bodmanstr. 22, 87439 Kempten, Germany; 4 Division of Thoracic Oncology, Department of Surgery, University Medical Center Mannheim, University of Heidelberg, Theodor-Kutzer-Ufer 1-3, 68135 Mannheim, Germany; 5 Clinical Cooperation Unit Nuclear Medicine, German Cancer Research Center, Heidelberg, Germany; 6 Department of Medical Oncology, University of Tübingen, Tübingen, Germany; 7 Division of Surgical Oncology and Thoracic Surgery, Department of Surgery, University Medical Center Mannheim, University of Heidelberg, Theodor-Kutzer-Ufer 1-3, 68135 Mannheim, Germany

**Keywords:** thymic carcinoma, thymoma, therapy, receptor tyrosine kinase, sunitinib, metastasis

## Abstract

**Background:**

Thymic carcinoma (TC) is a rare aggressive tumour. Median survival with current treatments is only 2 years. Sunitinib is a multi-targeted tyrosine kinase inhibitor that has shown benefit in various other cancers.

**Methods:**

Laboratory analyses of snap-frozen tumour tissues were performed to detect activation and genetic mutations of receptor tyrosine kinases (RTKs) in TC samples. On the basis of molecular analyses showing activation of multiple RTKs in their tumour, four patients with metastatic TCs refractory to conventional therapies were treated with sunitinib according to standard protocols.

**Results:**

RTK analysis in three of the patients showed activation of multiple RTKs, including platelet-derived growth factor-*β* and vascular endothelial growth factor 3. Mutations of *EGFR*, *c-KIT*, *KRAS*, and *BRAF* genes were not found. Administration of sunitinib yielded a partial remission (lasting 2 to 18+ months) according to the RECIST criteria in three patients and stable disease with excellent metabolic response in 18F-FDG-PET in another one. The overall survival with sunitinib treatment ranges from 4 to 40+ months. Withdrawal of the drug in one patient prompted rapid tumour progression that could be controlled by re-administration of sunitinib.

**Conclusions:**

Sunitinib is an active treatment for metastatic TC. A panel of molecular analyses may be warranted for optimal patient selection.

Thymic carcinoma (TC) is a rare aggressive tumour of the thymus (Eng *et al*, 2004). It affects men nearly twice as often as women, across a wide age range (Travis *et al*, 2004). Patients often initially present with cough and chest pain. Further work-up usually reveals a mediastinal mass. Histologically, TCs are rather heterogeneous and resemble tumours found in other organs (Travis *et al*, 2004). The prognosis is generally poor, and the majority of patients develop recurrences. Lymph nodes, lungs, liver, and bones are common sites for metastases. Patients with TC have a median survival of 2 years (Eng *et al*, 2004).

The optimal management of TCs remains an unresolved question, because of their rarity and aggressiveness (Kurup and Loehrer, 2004). Complete surgical resection substantially improves survival rates, but this is not always possible, because of invasion of surrounding structures or metastasis (Wright and Kessler, 2005). Adjuvant radiation with 40–70 Gy often follows surgical resection, but a survival advantage for radiotherapy has not been clearly demonstrated (Korst *et al*, 2009). Chemotherapy with regimens containing cisplatin have often yielded partial remissions (Evans and Lynch, 2005), but some patients do not respond at all and most patients do not achieve long-lasting remission. Thus, many patients eventually cannot be helped by any currently available treatments, and they succumb to the tumour's rapid progression.

Targeted molecular therapy is a new paradigm in cancer treatment, in which drugs selectively interfere with molecules considered important in oncogenesis. Whereas conventional chemotherapy aims to kill all proliferating cells including tumours, targeted molecular therapy aims to disrupt cancer-specific signalling pathways involved in tumour growth and proliferation (Faivre *et al*, 2006). Compared with the toxicity of chemotherapy, targeted molecular therapies seem to be relatively tolerable (Rutkowski and Ruka, 2009). There are multiple types of targeted molecular therapies, but among them, multi-target tyrosine kinase inhibitors have received particular attention. Tyrosine kinases regulate important cell functions, including survival, differentiation, and proliferation (Faivre *et al*, 2006). When mutated or overexpressed, they have key roles in many cancers: increasing tumour cell growth and proliferation, inducing resistance to apoptosis, and promoting angiogenesis and metastasis.

Sunitinib (Pfizer, New York, NY, USA) is a potent multi-target tyrosine kinase inhibitor, designed to selectively block the intracellular receptor-binding sites of several tyrosine kinases: vascular endothelial growth factors 1–3 (VEGF1–3), FMS-like tyrosine kinase 3 (FLT3), stem cell growth factor (c-KIT), platelet-derived growth factors-*α* and -*β* (PDGF*α*–*β*), colony-stimulating factor 1 (CSF1), and the ‘RET’ receptor for glial-derived neurotrophic factors (Faivre *et al*, 2007). Inhibition of these tyrosine kinase receptors is believed to ultimately result in tumour regression primarily through anti-angiogenic effects and also through direct tumour cell apoptosis. In clinical trials, sunitinib has been reported to be beneficial against metastatic clear-cell renal carcinoma (Motzer *et al*, 2007), gastrointestinal stromal tumour (Demetri *et al*, 2006), and advanced pancreatic neuroendocrine tumours (Kulke *et al*, 2008). The most commonly reported adverse events (such as fatigue, diarrhoea, nausea, anorexia, skin discolouration, and hand–foot syndrome) have been reported as relatively tolerable (Faivre *et al*, 2007; Porta *et al*, 2007).

For TC, there have only been a few peer-reviewed journal papers on any of the new targeted molecular therapies. We previously reported a single case in which imatinib led to temporary regression of liver metastases and stabilisation of the primary TC (Strobel *et al*, 2004). Another case report briefly mentioned the unsuccessful last-resort use of imatinib in a paediatric patient (Kertesz *et al*, 2007). One paper reported a partial response of a malignant thymoma to the abl/src kinase inhibitor dasatinib (Chuah *et al*, 2006).

In addition, there have been two case reports on the successful usage of sorafenib in heavily pre-treated, chemotherapy-resistant metastatic patients (Bisagni *et al*, 2009; Li *et al*, 2009).

To our awareness, there have been no reports of using sunitinib against any tumours of the thymus. The aim of this paper is to report the clinical outcomes and laboratory findings from our initial usage of sunitinib in metastatic TCs.

## Methods

### Patients

Tumours were classified according to criteria of the WHO classification (Travis *et al*, 2004). Patients with metastatic TCs refractory to conventional treatment were considered eligible for treatment with sunitinib and inclusion in this report. All patients provided informed consent for treatment and reporting of their data.

### Treatment

Patients were treated by interdisciplinary teams experienced in the application of tyrosine kinase inhibitors. Dose selection of sunitinib was dependent on tumour load, tumour progression slope, and expected toxicity profile related to the patients’ co-medication and medical history, not always following the 50 mg per day, 4/2 regimen (Faivre *et al*, 2007) (4 weeks on, 2 weeks off treatment, see details below). Toxicity and response were closely monitored, to adjust the dosage or cycle length if required. Other concurrent therapies were used whenever indicated. Tumour response was assessed according to the revised RECIST criteria (Eisenhauer *et al*, 2009). Further medical history and adverse events were charted.

### Laboratory analyses

Native tumour samples were available from patients A–C and kept snap frozen and stored at −80°C for molecular analyses. Paraffin sections were stained by a standard avidin–biotin peroxidase technique. Primary antibodies included CD5, c-KIT, and ki67.

The Human Phosopho-Receptor Tyrosine Kinase Array Kit (R&D Systems, Wiesbaden, Germany) was used according to the manufacturer's instructions to simultaneously detect the relative tyrosine phosphorylation levels of 42 different receptor tyrosine kinases (RTKs), as described previously in more detail (Ball *et al*, 2007).

Both DNA and RNA were extracted and amplified by PCR using commercial kits (QiAamp DNA FFPE and RNeasy FFPE; Qiagen, Hilden, Germany) according to the manufacturer's instructions. *c-KIT* (exons 9, 11, 13, 17), *EGFR* (exons 18–21), *KRAS* (exon 2), and *BRAF* (V600E) were sequenced according to standard procedures (details available upon request).

## Results

### Laboratory findings

In addition to histomorphological criteria, CD5 and c-KIT were strongly expressed in all cases, providing very strong evidence that these were indeed primary TCs (Travis *et al*, 2004).

**Figure 1:**
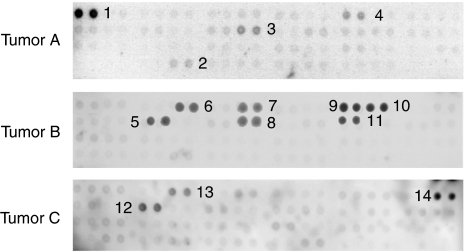
Phospho-protein arrays of tumour samples from patients A–C with 42 spotted receptor tyrosine kinases (signal indicates activated protein). Tumour sample of patient A shows strong activation of EGFR (1) and weaker signals for EphB2 (2), KIT (3), and the insulin receptor (4). Tumour sample of patient B shows moderate signals for RON (5), Erb4 (6), FGFR2 (7), KIT (8), and strong signals for insulin receptor (9), IGF1R (10), and RET (11). Tumour sample of patient C shows moderate signals for RON (12), Erb4 (13), and Tyro3/Dtk (14).

Case A mainly showed activation of the EGFR, with a very weak signal for the insulin receptor (IR), KIT, and Ephrin B2 (Figure 1). Case B showed strong activation of several RTKs, including IR and insulin-like growth factor receptor 1, fibroblast growth factor receptor 2, KIT, Erb4, and RET (Figure 1) Case C showed activation of ErB4, Tyro3/Dtk, and RON (Figure 1).

Using PCR and direct sequencing of the resulting amplification products, no mutations were found for *c-KIT*, *KRAS*, *BRAF*, or *EGFR* (not shown).

### Patients

The patients’ basic sociodemographic and clinical characteristics are presented in Table 1. Three patients in Table 1 were metastatic at the initial diagnosis. The patients’ earlier history of cancer and treatment is summarised in Table 2. In brief, all patients had already reached a point of having metastases not responding to established treatment options, before they were started on sunitinib.

### Clinical outcomes

**Figure 2:**
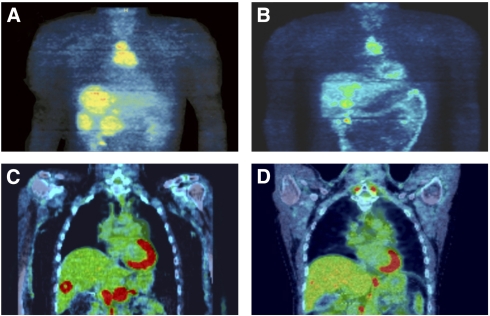
PET scans of the primary tumour and liver metastases from patient A. (**A**) Baseline just before initiating sunitinib. (**B**) After 6 months (in 4 2007) sunitinib. (**C**) Three months after withdrawal of sunitinib (10 2009) showing a highly enriched index metastasis. (**D**) The same lesion was undetectable 2 months after re-administration of sunitinib (12 2009).

For patient A, sunitinib was administered for 2 years, 9 months at a dose of 50 mg per day, taken continuously (never cycled with ‘off ’ periods). After just 2 weeks, the patient's abdominal pain disappeared. The 18F-FDG-PET scans showed reduced glucose metabolism of all liver metastases after 6 weeks in comparison with pre-treatment values. After 6 months, the vessel density calculated by FDG-PET of the liver metastases was reduced to <20% of their pre-sunitinib levels (Figure 2A–B). As the primary tumour seemed to respond less to the treatment than the liver metastases, concurrent radionuclide treatment with DOTATOC was administered during the 4th–11th months of sunitinib. Thereafter, the vessel density of the metastases was still very low, and the metastases were classified as stable disease according to RECIST (Eisenhauer *et al*, 2009). After 22 months of sunitinib treatment, the liver metastases started to progress again with new lesions; hence, selective infusional radiotherapy was started, resulting in a PR. Another 8 months later (4 2009), the primary tumour was completely resected, followed by 54 Gy irradiation of the mediastinum. As all known tumour lesions seemed to be under control, sunitinib was discontinued in 7 2009. Three months later, PET-CT showed massive progression of the hepatic metastases (Figure 2C) and lymph node metastases at the coeliac axis. Sunitinib was re-administered at 50 mg per day in 10 2009. Two months later, a control PET-CT showed marked regression of all lesions, corresponding to PR (Figure 2D).

**Figure 3:**
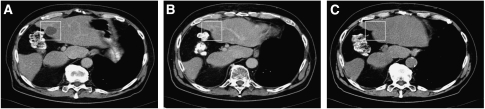
CT scans of a representative liver metastasis from patient B. (**A**) Liver metastasis at baseline just before initiating sunitinib. (**B**) Liver metastases after 6 months sunitinib. (**C**) Liver metastases after 18 months sunitinib.

Patient B presented with asymptomatic liver metastases. Sunitinib was started at the registered 50 mg per day, 4/2 regimen. The metastases were still progressing after one cycle but developed a PR after another three cycles (Figure 3A and B). After four cycles of 4/2, the cycling schedule was slowed to 4/4 because of side effects during the ‘on’ phase, which resolved during the ‘off phase’. After 10 months of therapy, the cycling was slowed to 4/6 for another two cycles, and further 4/8 dosage thereafter. At all follow-up visits since the fifth cycle of sunitinib until the present time (i.e., for 18 months), the liver metastases were still in good partial remission, and there has been no evidence of relapse of the primary tumour (Figure 3C).

**Figure 4:**
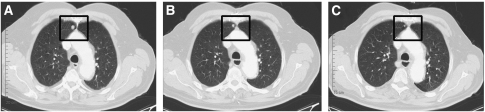
CT scans of an index lung metastasis from patient C. (**A**) Lung metastasis (square) at baseline just before initiating sunitinib. (**B**) Lung metastasis after 3 months sunitinib. (**C**) Lung metastasis after 7 months sunitinib.

Patient C presented with several metachronous asymptomatic lung metastases. Owing to his significant co-medication for severe NYHA III problems and hypertension, sunitinib was started at a dose of 25 mg per day. As the medication was well tolerated, the dose was escalated to 37.5 mg per day after 4 weeks and antihypertensive medication was intensified. The CT controls at 7 months showed partial remission of his lung metastases (Figure 4A–C). As the patient later on developed leukopenia with a WBC of <1800 cells per ml and anaemia, sunitinib was again decreased to 25 mg per day after 9 months. After 14 months under sunitinib (2 2010), the patient is still in PR.

In patient D, who suffered from a particularly aggressive, widely disseminated tumour at initial presentation, symptoms (such as shortness of breath) improved after 2 weeks of sunitinib with 50 mg per day, 4/2 dosing. Restaging after 6 weeks showed a PR of the primary tumour and lung metastases. However, this was soon followed by rapid tumour progression, to which the patient succumbed 4 months after the start of sunitinib and 16 months after initial diagnosis.

### Side effects

Commonly reported side effects of sunitinib were also observed in our patients. Nausea was present in three patients. Hypertension and fatigue were present in patients A and C. Weakness and oedema were present in patients C and D. Patient D had some minor, non-relevant electrophysiological cardiac alterations, but no hypertension or haematological complications. Grade 2/3 hand–foot syndrome in patient B lead to prolonged ‘off’ phases in the treatment cycles to allow for recovery.

## Discussion

Thymic carcinoma is a rare tumour that is difficult to treat in advanced stages. Cisplatin-based multi-agent chemotherapy can be beneficial, but often the responses do not last long. There is currently no other established second-line treatment for TC. Targeted molecular therapy could open new therapeutic options in these patients.

Our initial experience shows that sunitinib seems promising for patients with metastatic TC. The rationale for choosing this drug came from our observation that three of the available tumour samples showed simultaneous activation of multiple RTKs. Hence, it seemed reasonable to interfere with several of these processes concurrently to prevent tumour escape mechanisms through alternative signalling pathways (Faivre *et al*, 2006; Potapova *et al*, 2006).

The new targeted molecular therapies are clinically effective because they have been designed to disrupt the cellular signalling pathways involved in various aspects of oncogenesis, such as growth signal transduction, cell invasion, evasion of apoptosis, and metastatic dissemination. Sunitinib was originally designed and developed for its high potency against VEGFR2 and PDGFR-*β* (Burstein *et al*, 2008), and it is known to block the intracellular ATP-binding sites of several other RTKs: VEGF1, VEGF3, FLT3, c-KIT, PDGF*α*, CSF1, and RET (Faivre *et al*, 2007). Thus, on the basis of past scientific knowledge (Chow and Eckhardt, 2007) as well as our clinical observations and laboratory results, it seems plausible that the predominant mechanism of action of sunitinib in our patients was anti-angiogenesis, even though its main known targets were not prominently activated in the tumour samples studied herein. However in case A, the patient's PET scans showed a rapid and substantial reduction in tumour vessel density after the initiation of sunitinib, consistent with an anti-angiogenic mechanism of action. Many other RTKs also promote angiogenesis indirectly (Al-Nedawi *et al*, 2009) and thus could account for this finding. Taken together, the clinical observations, laboratory analyses, and previous molecular knowledge on sunitinib are consistent in supporting the view that sunitinib's effectiveness in these cases was due primarily to anti-angiogenesis.

Nonetheless, sunitinib may have had other mechanisms of action, in addition to anti-angiogenesis or instead of it. Sunitinib blocks many more RTKs than the literature or the manufacturer currently discusses. Furthermore, our array only tested for 42 of ∼300 RTKs; hence, many other RTKs may have been activated in our patients. Further clinical studies will be required to analyse the underlying mechanisms more thoroughly.

Although our findings are limited by the small sample size, sunitinib seems to be a particularly good choice of second-line therapy for patients with metastatic TC. The tumours reported in this study were histologically and immunohistochemically comparable and representative for a majority of TCs in the broader population. Moreover, in line with previous reports (Tsuchida *et al*, 2008; Yoh *et al*, 2008), there were no mutations of *c-KIT*, *KRAS*, *BRAF*, or *EGFR*; therefore, apparently the effectiveness of sunitinib does not depend on such abnormalities.

Our initial observations suggest that sunitinib (and possibly also other multi-kinase inhibitors such as sorafenib (Bisagni *et al*, 2009)) may be more effective against TC than the disappointing results reported in the grey literature for single-target molecular therapies, such as gefinitinib (Kurup *et al*, 2005), erlotinib (Bedano *et al*, 2008), and imatinib (Salter *et al*, 2008). These earlier results seem plausible in the light of the broad and rather heterogeneous spectrum of activated RTKs in the few samples described in this study.

Our ‘index’ patient A provides very strong circumstantial evidence that sunitinib was able to control his disease over more than 3 years and even at relapse of sunitinib pre-treated metastases. Similar favourable findings in two other patients (B+C) indicate that sunitinib may be able to block tumour escape mechanisms and may be a promising option for long-term treatment. Patient D (with an unusually aggressive tumour) obtained a temporary partial remission, which we estimate prolonged her life by about 1–3 months. It is noteworthy that although tumours from patients A–C were ‘classical’ squamous cell TCs, the tumour from patient D was undifferentiated and had unusually high mitotic counts, prompting us to rule out EBV association or a so-called ‘carcinoma with t(15;19) translocation’ (French *et al*, 2003) at initial presentation by respective molecular techniques.

In conclusion, sunitinib could be a promising new treatment option for TCs. Compared with many other tumours such as advanced prostate cancer (Dror Michaelson *et al*, 2009), metastatic colorectal cancer (Saltz *et al*, 2007), metastatic breast cancer (Burstein *et al*, 2008), or advanced non-small-cell lung cancer (Socinski *et al*, 2008), in which sunitinib was either inefficient or had only weak benefits were overridden by serious risks, TC seems to be one of the types of cancer that is responsive to sunitinib. It will be important to investigate whether patients with malignant thymomas may also benefit from this drug.

## Figures and Tables

**Table 1:** Patient sociodemographic and clinical characteristics

**Case**	**Sex**	**Age at diagnosis (years)**	**Stage (initial diagnosis)**	**Histopathology**	**Stage (start of sunitinib)**	**Tumour grade**
A	M	35	IVb	Squamous cell	IVb	2
B	M	69	IVa	Squamous cell	IVb	2
C	M	77	II	Squamous cell	IVb	2
D	F	28	IVb	Undifferentiated	IVb	3

**Table 2:** Previous treatment and result of sunitinib therapy

**Patient**	**Previous treatment**	**Extent of tumour at start of sunitinib**	**Treatment result**	**Survival (sunitinib)**
A	Systemic chemotherapy, PR for 3 months, imatinib 400/800 mg PD	Primary tumour liver metastases (60% HR) axillary, supraclavicular, and coeliac lymph node metastases	SD^a^ of primary tumour and hepatic metastases, excellent metabolic response (FDG-PET), PR^a^ of coeliac lymph node metastases	OS 40+ months
B	Primary tumour resection, radiation to mediastinum DFS 4 years, Systemic chemotherapy for liver metastases, PD	Liver metastases	PR^a^	PFS 18+ months
C	Primary tumour resection, DFS 14 months	Bilateral lung metastases	PR^a^	PFS 14+ months
D	Systemic chemotherapy, radiation of retinal metastases, PR for 6 months	Primary tumour, multiple bone and lung metastases (all PD)	PR^a^ for 2 months	OS 4 months

Abbreviations: PR=partial remission; PD=progressive disease; HR=hepatic replacement; SD=stable disease; OS=overall survival; DFS=disease-free survival; PFS=progression-free survival.

a Acc to RECIST (Response Evaluation Criteria In Solid Tumors) criteria.
